# Kounis syndrome in a patient following AstraZeneca coronavirus disease 2019 vaccination: a case report

**DOI:** 10.1186/s13256-023-04022-9

**Published:** 2023-06-28

**Authors:** A. M. Farook, D. Priyankara

**Affiliations:** 1 Critical Care Medicine, National Hospital of Sri Lanka, Colombo, Sri Lanka; 2 National Hospital of Sri Lanka, Colombo, Sri Lanka

**Keywords:** Case report, Kounis syndrome, Anaphylaxis, Allergic angina syndrome

## Abstract

**Introduction and importance:**

Kounis syndrome, also known as allergic angina syndrome, is defined as the occurrence of an acute coronary syndrome concomitantly with a hypersensitivity reaction. It is a very important diagnosis and missing it may be fatal to the patient. This is a medical emergency, and immediate treatment should be initiated. The treatment of Kounis syndrome is challenging because treatment of either acute coronary syndrome and hypersensitivity reaction can lead to the worsening of the other injury. This case is the first reported case of Kounis syndrome following coronavirus disease 2019 vaccination in Sri Lanka according to our knowledge.

**Case presentation:**

We discuss a 54-year-old female Sri Lankan patient who developed Kounis syndrome following Oxford AstraZeneca COVID-19 vaccination. The patient initially developed anaphylaxis following the AstraZeneca COVID-19 vaccine and subsequently developed acute coronary syndrome secondary to anaphylaxis. The patient was treated appropriately and eventually recovered from her condition.

**Conclusion:**

This syndrome should be suspected when there is a concurrent acute coronary syndrome with allergic reactions. This is an often under- or misdiagnosed condition, and physicians should be educated about it. Caregivers should be aware of its pathophysiology, as treatment of either of the two may worsen the other injury.

## Introduction

Kounis syndrome is not a rare, but often under- and misdiagnosed, disease; thus, leading to inappropriate treatment. It has rarely been discussed and reported in the literature. The incidence of this syndrome among all patients with allergies was estimated to be 19 per 100,000. It has been reported in every geographical location, age group, and every race.

## Case presentation

Our patient is a 54-year-old healthcare worker of Sri Lankan origin with a history of hypertension, ischemic heart disease stable angina, and bronchial asthma on inhalers. She also has a significant history of allergies, including multiple drug allergies and an episode of contrast-induced severe anaphylaxis 2 years previously. She was administered the AstraZeneca coronavirus disease 2019 (COVID-19) vaccine on the 22 December 2021 at 9:00 am and was under observation. Fifteen minutes later at 9:15 am, she developed a severe anaphylactic reaction with generalized urticaria, accompanied by shortness of breath, nausea, and severe vomiting. Her vitals were: temperature of 37.2 °C, pulse rate of 120 beats per minute, blood pressure of 80/50 mmHg, respiratory rate of 28 breaths per minute, SpO_2_ of 90%, and she had diffuse rhonchi in the lungs bilaterally. She was in anaphylactic shock. Immediately intramuscular 1:10,000 adrenaline was administered. Due to poor response, repeated doses of intramuscular adrenaline was given. She was also commenced on oxygen inhalation and antiallergy treatment (intramuscular promethazine hydrochloride 25 mg, intravenous hydrocortisone). Fluid resuscitation was done with normal saline boluses. Despite treatment, her symptoms were slow to improve. Arterial blood gas showed severe metabolic acidosis and the patient was started on an epinephrine infusion. Symptoms of the patient gradually improved with this. She was transferred to our hospital on the same day at 10:00 am for further management and observation of the recurrence of anaphylaxis. On arrival at the intensive care unit (ICU) at 10:15 am, she was clinically stable. Fifteen minutes after arrival at 10:30 am, she complained of severe retrosternal chest pain. Her vitals were: temperature of 37.0 °C, pulse rate of 115 beats per minute, respiratory rate of 26 breaths per minute, and blood pressure of 78/50 mmHg. Electrocardiogram (ECG) showed sinus rhythm, ST-segment elevation in leads I, II, and aVL ST-segment depression and T-wave inversion in leads III and V1–V4; her serum cardiac troponin I (cTnI) level was 17.61 ng/ml (< 0.5), and IgE levels were elevated at 895.7/IU/ml. The COVID-19 nucleic acid test (throat swab) was negative. Bedside, cardiac echo showed left ventricular global systolic dysfunction with a left ventricular ejection fraction of 35%. Echocardiography showed wall motion abnormalities, namely a segmental hypokinesia in the lateral wall and inferior wall. The patient was administered dobutamine hydrochloride (15 µg/kg/minute) via intravenous pump to maintain blood pressure along with moderate rehydration therapy. Considering these clinical ECG and ECHO findings, the diagnosis of acute coronary syndrome concomitant with the anaphylactic reaction, in other words Kounis syndrome, was suspected. Antiischemic treatment was initiated. The cardiology team suggested medical/non-interventional management of her acute coronary syndrome as there is a history of severe anaphylaxis to contrast agents. As she had clinical features of heart failure, appropriate treatment was initiated for heart failure. Her antianaphylactic treatment was continued. With the above treatment, she gradually improved [1]. She was clinically stable and discharged on day 7 of illness. She was referred to a specialized cardiology clinic for review and follow-up in 1 month on discharge, but unfortunately she did not turn up for review.

### Diagnosis

The patient was diagnosed with type 2 Kounis syndrome. It was based on clinical, ECG, and echocardiographic findings. She initially had a typical anaphylactic reaction secondary to COVID-19 vaccination, which initially responded to anaphylaxis treatment, and then after a short while she developed a typical myocardial infarction with typical ECG changes, troponin rise, and echocardiographic findings, along with ongoing features of allergic reaction. The patient’s condition significantly improved after active treatment with antiallergic, anticoronary spasm, and antiplatelet therapy. This typical clinical picture was consistent with the diagnosis of type 2 Kounis syndrome [2].

## Discussion

The syndrome was first described in 1991 by Kounis and Zavras. Many environmental exposures and drugs can cause Kounis syndrome. It is not rare but underdiagnosed, and often misdiagnosed, leading to inappropriate treatment [3]. It has rarely been discussed and reported in the literature. It is estimated that the incidence of this syndrome among all patients with allergies was 19 per 100,000. It has been reported in every geographical location, age group, and race [4].

The mechanism of Kounis syndrome has not been well understood until now. Several pathophysiological mechanisms are described to explain acute coronary syndrome in anaphylactic reactions. The primary pathophysiological mechanism is the vasospasm of epicardial coronary arteries due to the release of inflammatory mediators during an allergic reaction. The release of inflammatory mediators such as histamine, leukotrienes, prostaglandins, and platelet activation factors from mastocytes and other inflammatory cells leads to anaphylactic activation. It leads to vasospastic angina and myocardial infarction. Another contributing factor in anaphylaxis, which may worsen the cardiac injury, is prolonged hypotension, especially in patients with critical coronary stenosis in whom hypotension provokes myocardial ischemia. Risk factors for Kounis syndrome include previous allergy, previous cardiovascular disease, hypertension, diabetes, and smoking. Several types of Kounis syndrome have been identified. Kounis syndrome type 1 (allergic vasospastic angina due to endothelial dysfunction) occurs in people without preexisting heart disease and is due to the allergic reaction triggering the release of vasospasctic cytokines, leading to angina and potentially dangerous arrhythmias. Kounis syndrome type 2 (allergic myocardial infarction) occurs in patients with preexisting asymptomatic or symptomatic coronary artery disease, in which the allergic reaction either triggers vasospasm or plaque erosion/rupture leading to myocardial infarction. Kounis syndrome type 3 (allergic stent thrombosis with occluding thrombus or stent restenosis) occurs in patients with cardiac stents and is characterized by stent thrombosis due to platelet activation secondary to the allergic reaction.

In our case, an anaphylactic reaction was triggered by the AstraZeneca COVID-19 vaccination. The risk of anaphylaxis (1 per 1,000,000 doses) is very low for many vaccines. However, anaphylactic reactions including Kounis syndrome, are potentially life-threatening and can occur immediately, usually within minutes, after exposure to a vaccine. Confirmed allergic reactions to vaccines are not frequently attributed to active ingredients, but rather to inactive ingredients or excipients, such as egg protein, gelatin, formaldehyde, thimerosal, and neomycin. Excipients are necessary and added to a vaccine for specific purposes, such as stimulating a stronger immune response, preventing contamination by bacteria, or stabilizing the potency of the vaccine during transportation and storage. There are two main potential allergenic/immunogenic excipients in COVID-19 vaccines, polyethylene glycol (PEG) and polysorbate 80. Polysorbate 80, an excipient in the AstraZeneca vaccine, is seen to trigger allergic reactions in those allergic to the compound. Polysorbate 80 is a more potent trigger of anaphylactic reactions compared to other excipients and can induce anaphylaxis leading to Kounis syndrome

Diagnosis of Kounis syndrome is based on clinical manifestations. Many cases are missed or underdiagnosed due to the unawareness of physicians. ECG will show infarction/ischemia suggestive of an acute coronary syndrome, most commonly in the inferior leads. Serum troponin, serum tryptase, and IgE will be elevated. A coronary angiogram is usually expected.

In managing Kounis syndrome, we should consider myocardial revascularization and treating allergic reactions. The difficulty lies in the fact that the treatment of either of the entities may worsen the other injury. There are no clear guidelines for treating Kounis syndrome up to now. Most of the recommendations are based on individual case reports [5].

Adrenaline is the medication of choice to relieve life-threatening anaphylaxis. However, the paradox is that it may aggravate myocardial ischemia and cause coronary vasospasm and arrhythmias, especially if administered intravenously. Thus, the intramuscular route of administering adrenaline is preferred in these patients. Both H1 and H2 antihistamines and ranitidine can be used to treat allergic reactions. Bolus use of these drugs can cause hypotension and compromise coronary flow. Thus, these drugs should be given slowly. Steroids are often used in allergic reactions, but may impair wound healing, causing myocardial wall thinning and cardiac aneurysms. Fluid resuscitation is an essential aspect of managing distributive shock. Patients with acute coronary syndrome risk pulmonary edema and hemodynamic instability, so fluid resuscitation should be done cautiously. Aspirin has a vital role in the management of angina, but it can induce or worsen anaphylaxis. In anginal pain, opioids have a vital role as an analgesic and anxiolytic, but their use may lead to mast cell degranulation, which may aggravate anaphylaxis. As such, they should be prescribed cautiously.

Primary percutaneous coronary intervention is the preferred reperfusion strategy in acute coronary syndrome. If it cannot be performed, thrombolysis should be considered.

## Conclusion

Kounis syndrome is a rarely recognized and underdiagnosed condition. Physicians should be aware of this condition and suspect it whenever patients present concurrently with angina and anaphylaxis. This is a medical emergency, and immediate treatment should be initiated for both conditions. The treatment of Kounis syndrome is challenging because treatment of either of the two can lead to the worsening of the other injury [5]. Treating this condition is a delicate balancing act, and extra precautions should be taken before initiating certain medications. There are no standard guidelines for the management of Kounis syndrome.

## Data Availability

All data generated or analyzed during this study are included in this published article and its additional files.
